# How to deal with burden of critical illness: A comparison of strategies in different areas of China

**DOI:** 10.12669/pjms.303.4600

**Published:** 2014

**Authors:** Pengcheng Liu, Liwen Jiang, Chengyue Li, Mei Sun, Alexander Rieger, Mo Hao

**Affiliations:** 1 Pengcheng Liu, School of Public Health, Fudan University, Shanghai, China.; 2 Liwen Jiang, School of Public Health, Fudan University, Shanghai, China.; 3 Chengyue Li, School of Public Health, Fudan University, Shanghai, China.; 4 Mei Sun, School of Public Health, Fudan University, Shanghai, China.; 5 Alexander Rieger, Medizinische University Innsbruck, Holzkirchen, Germany.; 6 Mo Hao, School of Public Health, Fudan University, Shanghai, China.

**Keywords:** Critical Illness Insurance, Comparison

## Abstract

***Objectives:*** This article aims to introduce, compare and analyze the design and development of Critical Illness Insurance systems in different parts of China under different social and economic conditions, to explain their characteristics and similarities. It may provide references to other countries, especially developing countries, to solve the problem of high medical costs.

***Methods:*** According to the methods in Comparative Economics, 3 areas (Taicang in Jiangsu, Zhanjiang in Guangdong, Xunyi in Shanxi) which are in high, medium and low socio-economic condition respectively were chosen in China. Their critical illness insurance systems were analyzed in the study.

***Results: ***Each system shares several common points, including coordinating urban and rural medical insurance fund, financing from the basic medical insurance surplus, and exploring payment reform and so on. But in the way of management, Taicang and Zhanjiang cooperate with commercial insurance agencies, but Xunyi chooses autonomous management by government. In Xunyi, multi-channel financing is relatively more dispersed, while funds of Taicang and Zhanjiang are mainly from the basic medical insurance surplus. The specific method of payment is different among these three areas.

***Conclusion: ***Because of the differences in economic development, population structure, and sources of funds, each area took their own mode on health policy orientation, financing, payment, coverage, and fund management to design their Critical Illness Insurance systems. This might provide references to other areas in China and other developing countries in the world.

## INTRODUCTION

Critical illness always leads to high disease burden.^1^ High medical expenses of critical illness treatment often influence a family a lot, especially for families with low income. High medical expenses, which is also called catastrophic health expenditure, may lead the patients and their families to “poverty for disease” or “back to poverty for disease”.^2^

China has been setting up its basic medical insurance systems over the past decades, including urban employees' basic medical insurance (UEBMI), urban residents' basic medical insurance (URBMI), and new rural cooperative medical system (NCMS).Though the basic medical insurance almost covered every person, its reimbursement level is far from reducing or eliminating the high medical burden, especially for the burden of critical illness treatment. With the high prevalence rate of diseases such as cancer, cardiovascular disease and so on, more and more people face the problems of “poverty for disease” and “back to poverty for disease”. The whole society calls for the establishment of a critical illness insurance system.

On August 30th 2012, National Development and Reform Commission (NDRC), Ministry of Health (MOH) and other four ministries and commissions of China issued “Guidance about implementation of urban and rural residents’ critical illness insurance system” (Guidance). The guidance points out that critical illness insurance based on the basic medical insurance is an institutional arrangement aiming at further ensuring high medical expenses of critical illness.^3^ It is of great significance for solving the problems of “poverty for disease” and “back to poverty for disease”, and reducing catastrophic health expenditure. In addition, it is a further improvement of the multi-level medical security system in China.

Now, all provinces in China are actively exploring appropriate medical security mode of critical illness insurance according to their local situation. Then, how does it go and what good experiences do we get?

***Development status of critical illness insurance in China*** ***: ***In fact, early in 2012, several provinces and cities have carried out the exploration about critical illness insurance system before the guidance was issued. Because of the difference in economic development, population structure and sources of funds, different areas in China take various modes of health policy orientation, financing, payment, coverage and fund management. Here we selected three representative areas (Taicang in Jiangsu Province, Zhanjiang in Guangdong Province, Xunyi in Shanxi Province) to analyze China’s critical illness insurance system.

***Taicang*** ***mode: ***Taicang city is located in the eastern coast of China, it is a developed county among the top ten counties, with less than one million people. The social and economic development level in Taicang is very high. As early as 2008,its medical security system had merged, UEBMI,URBMI and NCMS together, which lay a solid foundation to the establishment of Taicang’s critical illness insurance system.

The framework of Taicang’s critical illness insurance system is as follows: The local social security institution calls for bids to commercial insurance institutions, then buys commercial insurance with the surplus of social medical insurance fund. The management advantages of commercial insurance are also helpful to medical insurance. For the insured of social medical insurance, they are covered by the social medical insurance when their medical expenses are below its ceiling line. The part above the ceiling line (high medical expenses) are reimbursed by commercial insurance.^4^ This is called “critical illness reinsurance” in Taicang.

The orientation of Taicang’s critical illness insurance is an extension and supplement of the basic social medical insurance. It is dominated by the government, and undertaken by commercial insurance institutions. It is a supplementary insurance for critical illness under the guidance of policy.

In financing, Taicang established the urban and rural residents' basic medical insurance in 2008, which is managed by social security department of the local government together with UEBMI.^5^ The fund of critical illness insurance is mainly from the surplus of basic medical insurance fund. To be specific, the standard of insurance premium is 50 yuan per year for the insured covered by UEBMI, while 20 yuan for ones covered by urban and rural residents' basic medical insurance. Such secondary distribution aims to help the vulnerable groups, reflecting the equalization of critical illness insurance.

The critical illness insurance is mainly for the expense totally more than 10000 yuan in total after basic medical insurance reimbursement. Moreover, critical illness insurance reimbursement is based on medical treatment charge rather than classification of diseases. The starting pay line is 10000 yuan, medical treatment charge is reimbursed by different compensation ratio levels according to the total medical expense. Higher medical expense means higher compensation ratio, and there is no ceiling line to limit.

In management, Taicang takes the way of “to buy commercial insurance agency services with the medical insurance fund”, according to the new medical system reform scheme of central government of China. Cooperating with the people's health insurance company of China, Jiangsu branch, Taicang set up a joint office in response for managing its critical illness insurance, built hospital representative system in response for supervision. The joint office and hospital representatives supervise the medical process under the authorization of the government. To prevent induced consumption of medical institutions and medical resources waste, critical illness insurance takes total amount control for payment to hospitals.

***Zhanjiang mode: ***Zhanjiang is an underdeveloped city in western Guangdong province, with nearly 8 million people. Zhanjiang mode is similar with Taicang mode in some ways, because they both belong to a commercial insurance pattern. But Zhangjiang mode is more complicated. In Zhangjiang, its critical illness insurance is based on an insurance called “supplementary medical insurance for high expenses”. Here we first introduce what is “supplementary medical insurance for high expenses”.^6^ Since 2009, Zhangjiang gradually merged its NCMS with URBMI, set up a unified urban and rural residents’ medical insurance system which is managed by the local department of social security. Individual insurance premium of urban and rural residents’ basic medical insurance is set as two levels-20 yuan and 50 yuan according to people’s different ability to pay. Zhangjiang transfers 15% of urban and rural residents' basic medical insurance fund to buy “supplementary medical insurance for high expenses” from Zhanjiang branch of People's Insurance Company of China (PICC). This has greatly increased the reimbursement level. Based on this, in August 2012, Zhanjiang government established “critical illness allowance system”, improving Zhanjing mode furtherly.^7^

In financing, Zhangjiang set an unified line: 2 yuan for one person. Then, together with the surplus of urban and rural residents' medical insurance fund, critical illness allowance system fund is established. The specific compensation method is as follows: the starting line is 50000 yuan (after the basic medical insurance reimbursement), compensating 50% for the part above 50000 yuan but below 160000 yuan (the first level) or 180000 yuan (the second level) after reimbursement from the basic medical insurance and supplementary medical insurance for high expenses. For the part above 160000 yuan (the first level) or 180000 yuan (the second level), critical illness allowance system compensates 70%. The ceiling line is 250000 yuan (the first level) or 300000 yuan (the second level).In management, Zhanjiang furtherly cooperates with commercial insurance company to improve management efficiency. On one hand, it can make full use of the medical resources, on the other hand, it can effectively control medical costs. Meanwhile, critical illness allowance system holds the principles of small profit, consistently keeps a profit of only 3% a year. In way of payment, it takes “total amount control, month paid in advance, year-end settlement”.

***Xunyi mode: ***Xunyi county is a less developed area located in the northern Xianyang city, Shanxi province in western China, over 90% of the people are agricultural population. Firstly, the distinctive feature of Xunyi mode is the diversity of its financing. The fundings are from the government, enterprises and individuals, which reduce the pressure of financing a lot. Secondly, it does not connect with the commercial insurance companies.

In 2010, Xunyi county established a new rural cooperative medical allowance system for critical illness in the process of exploring solution to the problem of “poverty for disease” or “back to poverty for disease”, which provides the references of critical illness insurance for less developed areas of the whole country.

In financing, Xunyi county unifies UEBMI and NCMS together. They are run by management center of NCMS. The sources of the whole fund include financial investment of government, fund of new rural cooperative medical system, local civil affairs department and social enterprises.^8^ Personal financing standard is 300 yuan per person per year for ones involved in the medical insurance.5% of the whole fund surplus is used as the critical illness insurance fund. In coverage, critical illness insurance compensates specific diseases patients and ones whose single hospitalization costs are above 100000 yuan. In management, Xunyi county’s critical illness insurance does not choose commercial insurance companies, but managed by the new rural cooperative medical system. To ensure the fund safety and effective use of critical illness insurance, the center is required to report to the county government regularly and strengthen the fixed-point hospital inspection. What is more, the center should be audited by the audit office. In payment, global budget is chosen by new rural cooperative medical system center.

## DISCUSSION

According to the actual local situation, the above three critical illness insurance modes have made positive explorations in financing, the integration of urban and rural medical security systems, cooperation with commercial insurance organizations and payment reform. Each mode has its own characteristics. Here we will undertake comparative analysis about such three modes from aspects of fund pooling, financing, the operation and management, payment reform, to offer reference for other areas.

First of all, in terms of pooling fund, what these three modes have in common is co-ordinating urban and rural medical insurance fund, which has been agreed by all parties. It represents the future direction of development of medical insurance, which is also reflected in the critical illness insurance. Coordinating urban and rural medical insurance fund can largely solve the problem of insufficient funding, reduce management costs and highlight the social justice, which has laid a solid foundation for success of critical illness insurance. But for most other areas of China, NCMS and URBMI, respectively, is managed by the health sector and social security departments. As departmental interests are involved, there is a certain degree of difficulty merging NCMS and URBMI. This greatly increases the difficulty to replicate these modes in other areas of China. There is still no conclusion about which department is better for the management of the critical illness insurance pool fund, but co-ordinating the medical insurance funds suggests the future direction of health care, which is also supported by "guildance" of 2012. Of course, Taicang mode is very advanced for other underdeveloped areas of China because Taicang city coordinates funds of UEBMI, URBMI and NCMS. Because rights and responsibilities of insurance are closely linked, pooling fund is unfair for urban workers to a certain extent in that the annual payment of medical insurance for urban workers is much more expensive than urban and rural residents. One possible remedy is to rely on the strong local government finances to fill the gap.

In terms of sources of financing, the fund of the three models all come from the basic medical insurance pool fund surplus, which is closely related to local conditions. In Taicang city, for example, from the 1997 health care reform, average annual surplus of health insurance fund is around 8% and the cumulative surplus is over 800 million yuan.^9^ Thus, the critical illness insurance of Taicang city effectively improves the utilization efficiency of the health insurance fund, while meeting the needs of poor families. In addition, just in point of financing, Xunyi mode is easier to promote in economically underdeveloped areas, because its multi-channel financing pressure of the "four points" is relatively more dispersed, with little financial investment and the new rural cooperative medical insurance funds in the affordable range, mobilizing the power of the civil affairs departments and enterprises.

Secondly, in the way of operation and management, Taicang and Zhanjiang are "commercial insurance mode". They use commercial insurance agencies’ professional advantages and market mechanisms to improve the operating efficiency, service levels and quality of critical illness insurance. It should be emphasized that the key to success lies in the maintenance of the "guaranteed profit" principle by the commercial insurance companies. In Taicang city, for example, the social security department only allows commercial insurance companies to have about 4.5% of the revenue, and the revenue rate does not exceed 5%. If we promote this mode across the whole country, under the huge profits temptation, there is an uncertainty whether commercial insurance companies can adhere to this principle. 

Thirdly, in order to ensure the rational use and safety of critical illness insurance fund, payment reforms have been carried out in the three modes, all showing a "global budget" feature. Implementation of critical illness insurance policy may release some legitimate medical needs, but may also induce some of the unreasonable and excessive medical care seeking behavior, which results in the rapid growth of medical costs and threats the security and stability of health insurance fund. In order to improve the efficient use of health care costs and control the excessive growth of medical costs, during the implementation of critical illness insurance, payment reforms were carried out at the same time in these three modes. Specifically, Taicang city implements the payment methods of "global budget, performance evaluation, comprehensive settlement", Zhanjiang city implements "total amount control, month paid in advance, year-end settlement", and Xunyi county implements "inpatient fund global budget, outpatient fund global budget by clinic times, case-based pre-payment".

In summary, each place has established a corresponding critical illness insurance system in of its own model according to their regional economic level, stage of social development, health care needs and extent of insurance pooling fund. However, critical illness insurance is an innovative work. As Chinese regional social and economic situation is quite different. 2012 "guidance" just proposes principles and framework of the requirements in carrying out this work, Therefore, other regions need to fully consider their local conditions while carrying out their specific tasks, making sure that the level of guarantee must be compatible with the level of social economic development and medical spending, meanwhile, exploring appropriate cooperation mechanisms and modes.

## Authors Contribution

**PL, LJ**: data collection, research work and manuscript writing of this study. 

**CL,MS, AR:** data collection and editing of manuscript.

**PL, MH:** review and final approval of manuscript.

## Notes

***Source of funding:*** National Natural Science Foundation of China (71373004). Innovative Research Team in University from Ministry of Education of China [IRT0912], National Natural Science Foundation of China [71003025, 71303058, and 71373004], Humanities and Social Science Research Project from Ministry of Education of China [12YJCZH100]
